# Obstacles and facilitators to preventing fetal alcohol spectrum disorder: a qualitative study with general practitioners

**DOI:** 10.3389/fmed.2024.1280349

**Published:** 2024-02-29

**Authors:** Sébastien Leruste, François Baelen, Bérénice Doray, Thierry Maillard, Catherine Marimoutou, Michel Spodenkiewicz

**Affiliations:** ^1^Université de La Réunion—UFR Santé Site de l'IES, CHU de La Réunion, Saint-Pierre, France; ^2^INSERM CIC-EC 1410, CHU of Réunion Island, Saint-Pierre, France; ^3^Laboratoire EPI (Etudes pharmaco-immunologiques), UFR Santé, Université de La Réunion, CHU (Centre Hospitalier Universitaire) de La Réunion, Saint-Denis, France; ^4^Service de Génétique, CHU (Centre Hospitalier Universitaire) de La Réunion, La Réunion, Saint-Denis, France; ^5^TSAF (Troubles du Spectre de l’Alcoolisation Foetale), Fondation Père Favron, CHU (Centre Hospitalier Universitaire) de La Réunion, Saint-Pierre, France; ^6^Centre de Référence Anomalies du Développement et Syndromes Malformatifs Sud-Ouest Occitanie Réunion, Site Constitutif de La Réunion, Saint-Denis, France; ^7^SAF-OI, Saint-Louis, France; ^8^Moods Team, INSERM UMR-1178, CESP, Le Kremlin-Bicêtre, France; ^9^McGill Group for Suicide Studies, Douglas Mental Health University Institute, Department of Psychiatry, McGill University, Montréal, QC, Canada

**Keywords:** general practice, fetal alcohol spectrum disorder, prevention, Reunion Island, qualitative study

## Abstract

**Background:**

Fetal Alcohol Spectrum Disorders are the leading cause of non-genetic intellectual disability. The damage caused, although completely preventable, is irreversible and requires lifelong support. General Practitioners have an important role in the prevention of Fetal Alcohol Spectrum Disorders. However, evidence suggests that General Practitioners do not monitor systematically alcohol consumption among pregnant women.

**Objectives:**

The aim of this study was to understand the barriers and motivations of General Practitioners in the prevention of Fetal Alcohol Spectrum Disorders on Reunion Island.

**Methods:**

A qualitative research study was conducted by conducting semi-structured individual interviews with general practitioners. Participants were selected by random or snowball sampling. General practitioners who worked only in unscheduled care services were excluded from this study. After the interviews were transcribed, a verbatim analysis was performed according to the principles of grounded theory.

**Results:**

Twenty interviews were conducted by two researchers between November and December 2020. General practitioners expressed discomfort in addressing alcohol consumption and excessive drinking in women. They had inaccurate theoretical knowledge and a lack of practical experience with Fetal Alcohol Spectrum Disorders. They also showed little knowledge of the Fetal Alcohol Spectrum Disorders care pathway available on Reunion Island. Both patients and general practitioners expressed discomfort when discussing women’s alcohol consumption. Conflicting government policies were highlighted as alcohol promotion campaigns overshadowed Fetal Alcohol Spectrum Disorders prevention initiatives.

**Conclusion:**

General practitioners should be open and non-judgmental in their interactions with women and couples, with a focus on early detection and short-term intervention. General practitioners should be better educated about Fetal Alcohol Spectrum Disorders and have a clearer understanding of the Fetal Alcohol Spectrum Disorders care pathway.

## 1 Introduction

Alcohol is a known teratogen (1, 2). It disrupts neurogenesis and can lead to disorders of varying severity, collectively termed fetal alcohol spectrum disorders (FASDs), the most severe of which is fetal alcohol syndrome (FAS). This is the complete form including facial morphological features (short palpebral fissures, smooth philtrum, and thin upper vermillion.) severe growth retardation and psychomotor retardation. The worldwide incidence of FAS is estimated to be 1 in 1,000 births. FASD is 10 times more common, with an incidence of 1/100 (3). They are the leading preventable cause of neurodevelopmental disability and delay, representing a real loss of opportunity and requiring lifelong multidisciplinary support. Primary prevention is the cornerstone of FASD management (4).

Reunion Island is a French overseas territory of 860,000 inhabitants located in the western part of the Indian Ocean. The population of Reunion Island is characterized by significant social heterogeneity, with highly vulnerable and marginalized groups (5). These populations are at greater risk of alcohol consumption, particularly as Reunion Island has a low tax rate on the sale of locally produced rum (6). In terms of volume, rum accounts for more than a quarter of alcohol consumption in Reunion Island. In addition to this advantageous tax regime, the ubiquitous advertising campaigns with gondola heads at the supermarket entrances are a strong incentive to drink (6).

The problem of FASD has been known there since the 1970s (7). Several innovative structures have been created to prevent FASD: a FASD diagnostic center, a resource center, and a coordination and orientation platform for families and health professionals. In 2015, the local Regional Health Agency implemented an action plan with the objectives of conducting public awareness activities, training health professionals to detect alcohol consumption in pregnant women, and promoting access to diagnosis and evaluation of children with FASD (8). During the first evaluation of this action plan in 2019 (7), the Agency regretted the lack of compliance of the 845 general practitioners (GPs) in Reunion Island with the proposed training on FASD and the lack of effectiveness of the measures to identify alcohol consumption in pregnant women.

In France, GPs are front-line physicians, responsible for prevention, patient centered care, follow-up and coordination of the healthcare pathways in a holistic perspective. GPs have a primary role in prevention because of their long-term relationship with their patients and their families (8, 9). Even though French guidelines edited by the Ministry of Health recommendations encourage GPs to identify alcohol consumption in pregnant women and women of childbearing age, the pressure of consultation time, and due to the characteristics of their care activities, GPs regularly focus on curative care to the detriment of prevention and detection.

When a woman screens positive for alcohol consumption, the GPs provide a comprehensive examination of the patient with a focus on other metabolic risk factors but also on the consequences of this alcohol consumption (i.e., on mental health), and refer to more specialized services (i.e., addictology). They keep the coordination of the care provided by specialist physicians according to the problems identified as priorities.

The aim of this study was to understand the barriers and motivations of general practitioners in La Réunion in the prevention of FASD.

## 2 Materials and methods

### 2.1 Study design

We conducted a qualitative research study using semi-structured interviews based on the principles of grounded theory and following the COREQ guidelines (10). Two studies were conducted jointly by two researchers on the same sample of general practitioners. The first was conducted by Louise Delfarguiel (LD) on the detection of alcohol consumption in pregnant women and the identification of individuals with FASD, while the second was conducted by François Baelen (FB) and aimed to understand the obstacles and motivations faced by general practitioners in Reunion Island in the prevention of FASD.

### 2.2 Data sampling

The theoretical purposive sample was initially constructed based on diversity criteria, taking into account age, gender, practice location, and years of experience. Recruitment was random, supplemented by snowball sampling. Each physician was contacted individually by telephone or email. Only volunteers were selected to participate in the study. The criteria for non-inclusion were refusal to participate and physicians practicing complementary alternative medicine (CAM) or who exclusively practiced unscheduled care. Data collection was shared between two investigators, both general practitioners, both trained in the principles of qualitative research (11), who facilitated the interviews. One focused on the role of the family physician in the prevention of FASD and the other on the role of the family physician in the identification of children with FASD. Data collection consisted of individually recorded, semi-structured interviews. These interviews could be conducted either face-to-face in the family physician’s office or via videoconference. An interview guide was used to conduct the interviews (Appendix 1). This framework was designed beforehand by the two investigators under the guidance of the two research directors and then enriched and modified as the interviews progressed (Appendix n°1). The personal data (surname, first name, etc.) of the GPs were not recorded in order to limit the risk of identification. The interviews were numbered and transcribed anonymously. All audiovisual and voice recordings were destroyed at the end of the transcription process.

### 2.3 Analysis

After the verbatim transcription of the interviews, the analysis of the verbatim transcripts was carried out according to the principles of grounded theory (12, 13). The interviews were transcribed verbatim and coded using Nvivo® version 11.3 software, first in an open-ended manner and then progressively organized into axial coding. Data collection and analysis were conducted simultaneously, and hypotheses were constructed as the interviews progressed. As the analysis progressed, a categorization of these codings brought out concepts that were related. This analysis was subject to triangulation of data between interviewers to ensure the quality of coding. Discrepancies were addressed during meetings by a senior qualitative researcher Michel Spodenkiewicz (MS). Interviews were considered sufficiently numerous when a theoretical sufficiency of data was achieved and confirmed by the absence of new emerging ideas when additional interviews were conducted.

### 2.4 Ethics

Verbal consent was obtained. Anonymity of participation and interviews was ensured. No transcript was returned to participants. In accordance with current regulations, a request for compliance with the reference method (MR004) was submitted to the Commission Nationale de l’Informatique et des Libertés (CNIL), registered under number 2219961 v 0.

## 3 Results

Of the 31 GPs contacted, 20 agreed to take part in an interview (nine females and 11 males). All interviews were completed between November and December 2020. The mean length of the interviews was 18.7 min, the mean age of the participants was 47.8 years (median: 46.5), and the professional experience ranged from 2 to 39 years of practice (median: 17 years). A summary of participant characteristics is presented in Table 1.

**Table 1:** Characteristics of general practitioners interviewed during the study.

Interview	Gender	Age, (years)	Years practice	Duration of interview (min)
E1	M	30	2	21
E2	M	30	2	26
E3	M	60	30	22
E4	F	35	3	18
E5	M	62	31	32
E6	F	47	17	18
E7	F	41	12	25
E8	F	55	22	26
E9	F	64	39	38
E10	M	46	17	26
E11	M	64	35	12
E12	M	55	26	13
E13	F	47	19	17
E14	M	67	36	18
E15	M	31	4	20
E16	F	45	17	16
E17	M	59	30	26
E18	F	38	10	16
E19	M	42	15	23
E20	F	39	9	21

### 3.1 The general practitioner in the face of alcohol-related challenges

Numerous situations brought the family physicians to the problem of alcohol consumption. However, the assessment of this consumption was often approximate and subjective. Some used a quantification in “glasses of alcohol,” but none of the doctors interviewed used a standardized scale. Some physicians spontaneously mentioned pregnancy as a circumstance in which they asked about alcohol consumption. The importance of brief interventions and motivational interviewing was mentioned several times:

E1: “It’s motivational interviewing, so the patient has to find their own motivation, so I actually try to open doors as much as possible…”

On the other hand, many difficulties in addictology were underlined: the approach to alcohol remained sometimes difficult, especially compared to tobacco. Consultations related to an addiction problem were considered complex and time-consuming. Even specialized treatment was often unsatisfactory and sometimes discouraging in terms of results.

E19: “Maybe also because I have problems with the treatment, so if I feel that I have problems with something, I am not going to dig up something that I cannot handle.”

### 3.2 General practitioners and FASD: some knowledge, few diagnoses

All GPs had some knowledge of FASD. However, most of this knowledge was focused on the most severe form, FAS. This knowledge was still rather imprecise; most of the doctors interviewed stated that they had received little training on the subject, and none of them had been sensitized or recently trained on FASD. One notable finding was that all of the general practitioners emphasized that they had encountered few, if any, people with FASDs during their careers.

E10: “So obviously we must be missing some of them for sure, but…I do not really know…Or maybe it’s me who does not see it, I must not be trained enough, I do not recognize it.”

The few cases encountered were late diagnoses made by pediatricians or psychiatrists, at the time of school difficulties. Despite the existence of innovative structures in La Réunion (Resource Center, Diagnostic Center, Coordination and Orientation Platform), the doctors interviewed were not aware of their existence and of the care pathway in case of suspicion of FASD. Finally, some admitted their lack of interest in FASD.

### 3.3 Women and alcohol and the family doctor

Alcohol was seen as an even more taboo subject when it came to women. The pejorative view of society was thought to be responsible for a sense of shame and often a denial of alcohol consumption. Women’s drinking seemed to be more hidden than men’s and therefore more difficult for general practitioners to identify.

E15: “Honestly, I do not know the percentages and ratios of female/male addicts, but I think that women do not reveal themselves too much…”

Some doctors also recognized that there was denial on their side, as some were more likely to screen men for alcohol use than women.

E5: “But I’m a bit of an older generation and I think I tend to ask the question more often with men than with women. And that’s not good!”

Regarding the pregnant patient, most doctors said they systematically asked patients about possible alcohol consumption from the beginning of the pregnancy. Some asked later, especially when it was time to fill out the pregnancy record after several weeks of pregnancy. Some admitted that they did not do so systematically. Some of the doctors interviewed explained that they did very little follow-up with pregnant women. These doctors only intervened in the pregnancy on an *ad hoc* basis after an intercurrent problem. Some doctors mentioned the importance of pre-conception counseling in terms of prevention, especially with regard to alcohol consumption.

E18: “Maybe less so now that I think about it, but up to a certain point, when women decided to stop taking the pill, they came for a consultation…”

The important role of the doctor-patient relationship was emphasized, with the importance of being open to discussion, without judgment, without moralizing, and of being available, thus allowing the relationship of trust necessary to address a sensitive issue such as alcohol consumption.

### 3.4 The general practitioner in the face of contradictory policies

The efforts made in recent years to raise public awareness have been noticed by general practitioners.

E7: “Well, there are a lot of messages being broadcast these days…on the radio, on billboards…”

The message “no alcohol during pregnancy” was known to all participants, as was the idea of no minimum toxic dose. Patients were now considered by the participating general practitioners to be better informed about the consequences of alcohol consumption during pregnancy. Efforts to raise public awareness were considered essential by the participants. On the other hand, policies that facilitate access to alcohol on Réunion Island were highlighted: the virulence of advertising, the lucrative impact of the alcohol trade. Some even considered that government policies were responsible for the excessive consumption of alcohol by patients.

E18: “But I’m not going to feel guilty anymore, because if the government is not behind us…I’m not asking them to be behind us, but at least to avoid certain things, it’s complicated anyway.”

## 4 Discussion

### 4.1 Discussion of results

This study highlights the central role of primary care physicians in screening patients for alcohol use and, more broadly, in the prevention of FASD. The importance of early identification, brief interventions, and motivational interviewing was highlighted in this study. This finding is consistent with trends in the development of the profession (14). This task is fraught with many challenges, because although alcohol is an unavoidable problem in primary care, the physicians interviewed clearly expressed difficulties in dealing with alcohol and managing excessive consumption, which are widely documented in the literature (15). These deficits in addictology were also found in the evaluation of general practice residents at the end of their training (16). This suggests a lack of initial training especially on the screening procedures. Simulation based medical teaching on how to ask the right questions or to use the different screening tools in a real-life clinical setting could be helpful in overcoming this challenge (17). This training could use the supports designed by the Centers for Disease Control and Prevention.^1^

All general practitioners had some knowledge of FASD, mostly focused on FAS. Surprisingly, very few had encountered individuals with FASD during their careers. This finding contrasts with epidemiologic data (1). This result allows us to understand the lack of support from general practitioners. It is a vicious circle: the less doctors are trained and made aware of FASD, the less likely they are to identify a child with FASD early. The less they identify children with FASD, the less they will feel the importance of systematic upstream prevention. Despite efforts to raise awareness, there is still a stigma associated with female alcohol use. Several studies (18) have confirmed a more negative social view than for men. Alcohol consumption among women, including during pregnancy, is still underestimated. This study also suggests the interest of pre-conception counseling in terms of prevention. A study carried out in 2015 on 392 patients showed that only 15% of them had received a preconception consultation, with a strong predominance among primiparous women with a high socio-professional level (19). In France, recent studies suggest that the proportion of pregnancies supervised by general practitioners is decreasing: 19.3% of pregnancies supervised in 2016 compared with 23.8% in 2010 (20). At the same time, the percentage of pregnancies monitored by midwives increased from 16 to 25%. The obstetrician-gynecologist is still the main actor in pregnancy follow-up. Are general practitioners becoming less effective in their preventive role in pregnancy monitoring as they are asked to monitor fewer and fewer pregnancies? Some of the interviews conducted in this study seem to point in this direction.

The French government’s awareness campaigns “Alcohol and pregnancy, talk to your doctor”; “Zero alcohol during pregnancy” and the various means used to reach the population are recognized by the doctors interviewed. They consider that women are now well informed about the consequences of alcohol consumption during pregnancy. A 2015 qualitative research study points out that while almost all women agree that drinking alcohol during pregnancy is risky, half do not know exactly what the risks are (21). If heavy drinking is perceived as dangerous, the discourse is much more ambivalent when it comes to casual consumption. In Mété et al. (6), of a sample of 1,000 French people, 21% thought it was recommended to have a small drink from time to time during pregnancy (22). These results suggest that it is essential to continue these efforts to inform the population, as well as the individual prevention missions of the general practitioner. Finally, our interviews highlighted the ambivalence of the French government: the alcohol lobby is powerful and lucrative. The promotion of alcohol on the island of Réunion is massive, omnipresent and intensified during the holiday period (23). Alcohol, whether rum or beer, is linked to the Reunionese identity. Its taxation is even more advantageous than in metropolitan France.

### 4.2 Strengths and limitations

This was an original qualitative study in La Réunion. The methodology was developed according to the COREQ guidelines. The analysis respected the principles of grounded theory and data triangulation was carried out during coding to minimize interpretation bias. Theoretical sufficiency of the data was achieved after 15 interviews: the next five interviews only supported ideas already expressed by the participants and did not contribute any additional elements to the theory construction.

One of the weaknesses of this study was that the two interviewers had no previous experience in conducting a qualitative study and facilitating interviews. No data were obtained concerning neither GPs training on FASD, their experience in a systematic screening of FASD nor their own alcohol consumption. Training was provided by the dissertation advisors in advance to minimize internal researcher bias and investigator bias.

### 4.3 Perspectives and conclusions

As part of the action research to which this study belongs, a study will be conducted to implement prevention and detection measures in practice and measure their impact on general practitioners’ practices.

This study suggests that there are many opportunities for improvement. They support the strong investment of health actors, including users, in the fight against the problem of alcohol dependence and FASD. Several concrete actions could facilitate the prevention of FASD. Firstly, there is a need to continue the work started with public awareness campaigns. Secondly, general practitioners must be better involved in the prevention of FASD and, more generally, in the field of addiction with a better initial training and an increase in the value of consultations related to addiction problems seem relevant. The role of the general practitioner in the prevention of FASD is important, and his missions are early detection and brief interventions. The fight against the taboo of alcohol, which is even more pronounced for women, must be intensified. The question of alcohol consumption must be a recurring question, without relying on “one’s own impression.” It should be done systematically and as early as possible before the desire to become pregnant and during pregnancy: open questioning and systematic information on the risks associated with alcohol consumption. The importance of pre-conception counseling is recognized and efforts should be made to make it more systematic. Finally, Reunion Island could draw inspiration from other countries in terms of alcohol regulation policies: Norway and South Africa, for example, have banned all advertising of alcoholic beverages (20).

## Data availability statement

The raw data supporting the conclusions of this article will be made available by the authors, without undue reservation.

## Ethics statement

The studies involving humans were approved by a declaration to the university's data protection officer (RGPD). An anonymization step has been performed during the data processing in order to avoid the acquisition of personal data. In accordance with current regulations, a request for compliance with the reference method (MR004) was submitted to the Commission Nationale de l'Informatique et des Libertés (CNIL), registered under number 2219961v0. The consultation of a data protection committee was not required for this type of study. The studies were conducted in accordance with the local legislation and institutional requirements. The participants provided their written informed consent to participate in this study.

## Author contributions

SL: Conceptualization, Formal analysis, Methodology, Project administration, Resources, Supervision, Validation, Visualization, Writing – original draft, Writing – review & editing. FB: Conceptualization, Data curation, Formal analysis, Investigation, Methodology, Project administration, Visualization, Writing – original draft, Writing – review & editing. BD: Writing – review & editing. TM: Writing – review & editing. CM: Writing – review & editing. MS: Conceptualization, Formal analysis, Methodology, Project administration, Supervision, Validation, Writing – review & editing.

## Footnotes

^1^ https://www.cdc.gov/ncbddd/fasd/index.html

## References

[1] Fillaut T Hontebeyrie J Douguet F. Un pédiatre nantais « découvreur » du syndrome d’alcoolisation fœtale: le Dr Paul Lemoine (1917-2006). Psychotropes. (2017) 23:9–29. doi: 10.3917/psyt.231.0009

[2] Jones K Smith D. Recognition of the fetal alcohol syndrome in early infancy. Lancet Glob Health. (1973) 302:999–1001. doi: 10.1016/S0140-6736(73)91092-1 4127281

[3] Popova S Lange S Probst C Gmel G Rehm J. Estimation of national, regional, and global prevalence of alcohol use during pregnancy and fetal alcohol syndrome: a systematic review and meta-analysis. Lancet Glob Health. (2017) 5:e290–9. doi: 10.1016/S2214-109X(17)30021-9 28089487

[4] Nordmann R. Consommation d’alcool, de tabac ou de cannabis au cours de la grossesse. Bull Acad Nat Méd. (2004) 188:519–21. doi: 10.1016/S0001-4079(19)33780-X

[5] Obradovic I. (2020). Drogues et addictions dans les Outre-mer: état des lieux et problématiques. Paris: Observatoire français des drogues et les toxicomanies (OFDT).

[6] Mété D. Fiscalité des rhums traditionnels en outre-mer et santé publique: l’exemple de l’île de La Réunion [Taxation of traditional rums in French overseas territories and public health: the example of Reunion Island]. Rev Epidemiol Sante Publique. (2017) 65:443–52. doi: 10.1016/j.respe.2017.06.003, PMID: 29110959

[7] Lesure JF. Syndrome d’alcoolisme foetal à l’île de La Réunion. Nouv Press Med. (1980) 9:1708–10. PMID: 7193321

[8] La Réunion ARS (2017). Plan d’action régional de prévention du syndrome d’alcoolisation foetale (SAF)—Dossier de presse.

[9] Peadon E O'Leary C Bower C Elliott E. Impacts of alcohol use in pregnancy--the role of the GP. Aust Fam Physician. (2007) 36:935–9. PMID: 18043782

[10] La Réunion ARS (2020). Evaluation du processus du Plan d’action de prévention et de prise en charge de l’ensemble des troubles causés par l’alcoolisation foetale à La Réunion—Rapport final d’évaluation.

[11] Tong A. Consolidated criteria for reporting qualitative research (COREQ): a 32-item checklist for interviews and focus groups. Int J Qual Health Care. (2007) 19:349–57. doi: 10.1093/intqhc/mzm042, PMID: 17872937

[12] Aubin I. Introduction à la recherche qualitative. Exercer. (2008) 84:142–5.

[13] Lejeune C. Manuel d’Analyse Qualitative: Analyser Sans Compter ni Classer. Louvain-la-Neuve: De Boeck (2014).

[14] Beck F Guignard R Obradovic I Gautier A Karila L. Le développement du repérage des pratiques addictives en médecine générale en France. Rev DÉpidémiol Santé Publ. (2011) 59:285–94. doi: 10.1016/j.respe.2011.03.059, PMID: 21940125

[15] Andler R Cogordan C Pasquereau A Buyck J-F Nguyen-Thanh V. The practices of French general practitioners regarding screening and counselling pregnant women for tobacco smoking and alcohol drinking. Int J Public Health. (2018) 63:631–40. doi: 10.1007/s00038-018-1103-9, PMID: 29679105

[16] Djengué A Pham A-D Kowalski V Burri C. L’alcoologie et les futurs médecins généralistes français: Évaluation des connaissances, des pratiques et de la formation reçue en fin d’internat. Psychotropes. (2017) 23:89–109. doi: 10.3917/psyt.231.0089

[17] Al-Elq AH. Simulation-based medical teaching and learning. J Fam Community Med. (2010) 17:35–40. doi: 10.4103/1319-1683.68787, PMID: 22022669 PMC3195067

[18] Taschini E Urdapilleta I Verlhiac J-F Tavani J-L. Représentations sociales de l’alcoolisme féminin et masculin en fonction des pratiques de consommation d’alcool. Cah Int Psychol Soc. (2015) 107:435–61. doi: 10.3917/cips.107.0435

[19] Paradis S Ego A Bosson J-L. Preconception care among low-risk mothers in a French perinatal network: frequency of utilization and factors associated. J Gynecol Obstet Hum Reprod. (2017) 46:591–6. doi: 10.1016/j.jogoh.2017.05.002, PMID: 28526520

[20] Institut National de la Santé et de la Recherche Médicale, Direction de la Recherche, des Etudes, de l’Evaluation et des Statistiques (2016). Enquête Nationale Périnatale.

[21] Brahic J Thomas O Dany L. Alcool et grossesse: une recherche qualitative auprès de femmes enceintes. Cah Int Psychol Soc. (2015) 107:403–34. doi: 10.3917/cips.107.0403

[22] Cogordan C. (2017). Alcool et grossesse: connaissances et perceptions des risques, visibilité du pictogramme. Santé Publique France.

[23] Mété D (2015). Fédération régionale d’Addictologie de La Réunion. Lutter plus efficacement contre l’abus d’alcool à La Réunion.

